# Enhanced thermoelectric performance of Nb-doped SrTiO_3_ by nano-inclusion with low thermal conductivity

**DOI:** 10.1038/srep03449

**Published:** 2013-12-09

**Authors:** Ning Wang, Haijun Chen, Hongcai He, Wataru Norimatsu, Michiko Kusunoki, Kunihito Koumoto

**Affiliations:** 1 University of Electronic Science and Technology of China, State Key Laboratory of Electronic Thin Films and Integrated Devices, Chengdu, 610054, P. R. China; 2 Nagoya University, Graduate School of Engineering, Nagoya, 464-8603, Japan

## Abstract

Authors reported an effective path to increase the electrical conductivity while to decrease the thermal conductivity, and thus to enhance the *ZT* value by nano-inclusions. By this method, the *ZT* value of Nb-doped SrTiO_3_ was enhanced 9-fold by yttria stabilized zirconia (YSZ) nano-inclusions. YSZ inclusions, located inside grain and in triple junction, can reduce the thermal conductivity by effective interface phonon scattering, enhance the electrical conductivity by promoting the abnormal grain growth, and thus lead to the obvious enhancement of *ZT* value, which strongly suggests that, it is possible to not only reduce the thermal conductivity, but also increase the electrical conductivity by nano-inclusions with low thermal conductivity. This study will give some useful enlightenment to the preparation of high-performance oxide thermoelectric materials.

Thermoelectric (TE) energy conversion is a promising technology for both electricity power generation in harvesting wasted heat and electric cooling. The efficiency of TE devices is characterized by the dimensionless figure of merit, *ZT* = (*S*^2^*σ*/*κ*)T, where *S*, *σ*, *κ*, and *T* are the Seebeck coefficient, the electrical conductivity, the thermal conductivity, and the absolute temperature, respectively. To date, most of the discovered bulk materials with high *ZT* values exhibit thermal and chemical instability in air, and are composed of toxic, scarce or expensive elements^1^,^2^,^3^,^4^,^5^. Recently, various ceramic oxide thermoelectric (TE) materials have attracted widespread attention, because they are economical, environmentally-friendly, possess various chemical compositions, and consist of naturally abundant^6^,^7^,^8^,^9^,^10^,^11^,^12^,^13^,^14^. So far, among the *p*-type oxide thermoelectric bulk materials, the textured BiCuSeO has the highest *ZT* value, ~1.4 at 923 K^15^, which can meat the basic requirements of practical applications. Both *p*-type and *n*-type oxide materials are needed to construct an efficient thermoelectric device. However, among the *n*-type oxide thermoelectric bulk materials, even though Nb-doped SrTiO_3_ bulk ceramic has the highest *ZT* value so far^16^, but it is still very low, compared with *p*-type textured BiCuSeO, and thus urgently needs to improve.

In 2007, Dresselhaus *et al.* proposed that nanocomposite thermoelectric materials would offer a promising approach for the preparation of bulk samples with nano-sized constituents^17^. Since then, a variety of nanocomposites with higher thermoelectric properties than bulk materials have been prepared. Tang *et al.* prepared Bi_2_Te_3_ bulk materials with layered nanostructure by combining melt spinning technique with spark plasma sintering, and the maximum *ZT* value of 1.35 was obtained at 300 K^18^. Mi *et al.* prepared *n*-type CoSb_3_ nanocomposites by hot pressing the mixture of nanoscale and microsized CoSb_3_ powders, and the maximum *ZT* value reached 0.71 at 700 K^19^. Poudel *et al.* fabricated nano-sized bismuth antimony telluride bulk alloys by ball milling and hot pressing method, and the obtained maximum *ZT* value was 1.4 at 373 K^20^. Li *et al.* fabricated Yb_0.2_Co_4_Sb_12+y_ bulk materials with nanostructure by combining melt spinning technique with spark plasma sintering, and the maximum *ZT* value reached 1.26 at 800 K^21^. Li *et al.* also prepared *n*-type skutterudites In_x_Ce_y_Co_4_Sb_12_ with in-situ forming nano-sized InSb phase by a melt-quench-anneal-spark plasma sintering method, and the maximum *ZT* value of 1.43 was obtained at 800 K^22^. Lan *et al.* found that small nanosized particles located at the grain boundaries or embedded in larger particles could provide effective phonon scattering centers and thus reduced the phonon thermal conductivity efficiently^23^. Pei *et al.* found that there was an enhancement of *ZT* due to phonon scattering at the interfaces, when PbTe was nanostructured with large nanometer sized Ag_2_Te precipitates^24^. Ahn *et al.* prepared *p*-type PbTe-MTe (M = Cd, Hg) and found that meso-scale grain boundaries along with nanostructured precipitates play an important role in significantly reducing the lattice thermal conductivity^25^. Above-mentioned studies on thermoelectric materials with nanocomposite gave us some good inspirations.

Herein, we reported an effective path to enhance the thermoelectric performance of *n*-type oxide, Nb-doped SrTiO_3_ (SrNb_0.15_Ti_0.85_O_3_, Nb-STO), by yttria stabilized zirconia (YSZ) nano-inclusion with the low thermal conductivity. Our results show the *ZT* value of Nb-STO was enhanced 9-fold, due to the decreased thermal conductivity and meanwhile the increased electrical conductivity, pointing to a robust approach for high-performance *n*-type thermoelectric oxide.

## Results

**Figure 1:**
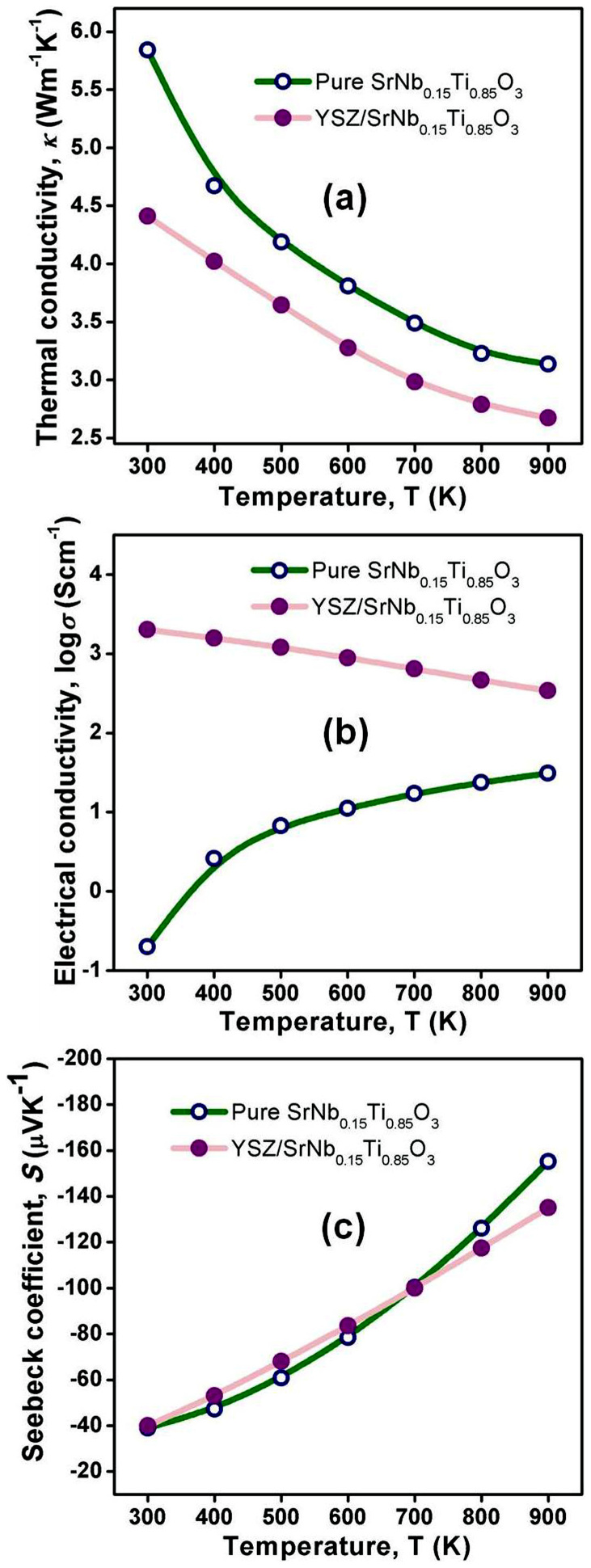
Temperature dependent thermal and electric transport properties of YSZ/Nb-STO composite. (a) thermal conductivity (*κ*), (b) electrical conductivity (*σ*), and (*c*) Seebeck coefficient (*S*).

Fig. 1 showed the temperature dependent thermal and electric transport properties of YSZ/Nb-STO composite, namely, the thermal conductivity (*κ*), electrical conductivity (*σ*), and Seebeck coefficient (*S*). Significantly, YSZ inclusion reduced the thermal conductivity (Fig. 1(a)), and meanwhile increased the electrical conductivity (Fig. 1(b). However, Seebeck coefficients were subjected to a very small impact by YSZ inclusion (Fig. 1(c)). The thermal conductivity and the electrical conductivity at 900 K were reduced ~15% and increased 10-fold, respectively.

**Figure 2:**
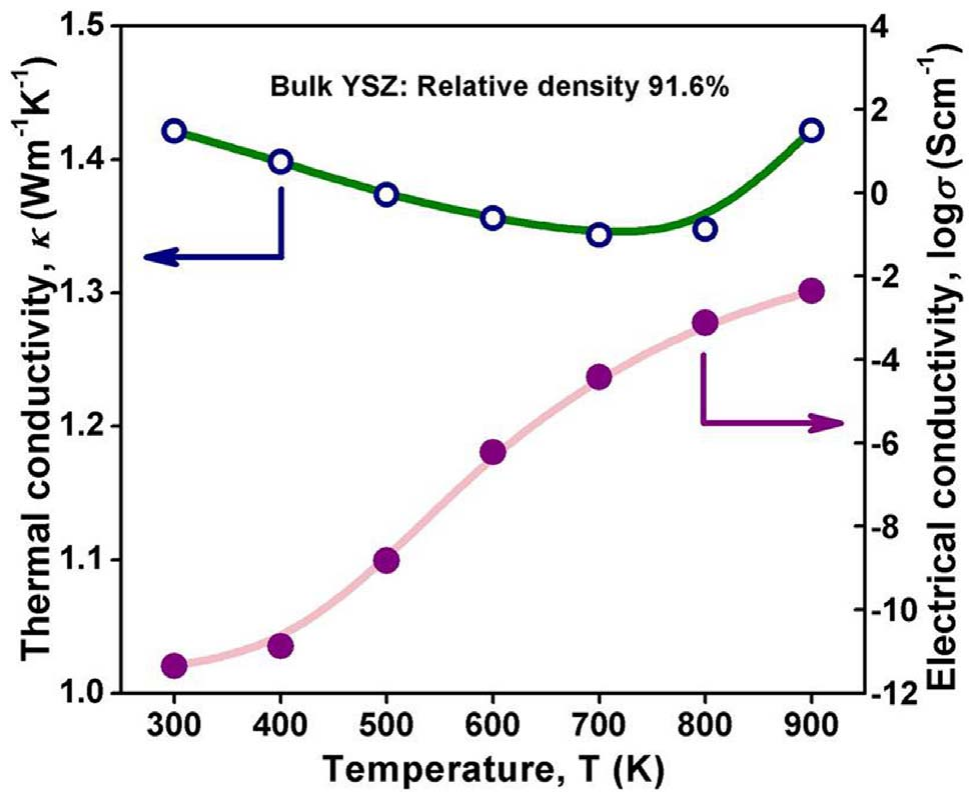
Temperature dependent electrical conductivity and thermal conductivity of pure YSZ bulk ceramic.

Fig. 2 showed the temperature dependent electrical conductivity and thermal conductivity of pure YSZ bulk ceramic, with the relative density of 91.6%, prepared by the conventional normal pressure sintering method at 1500°C for 3 h in an Ar atmosphere. The thermal conductivity and the electrical conductivity of YSZ bulk were much lower than those of pure Nb-STO ceramic showed in Fig. 1(a) and Fig. 1(b).

**Figure 3:**
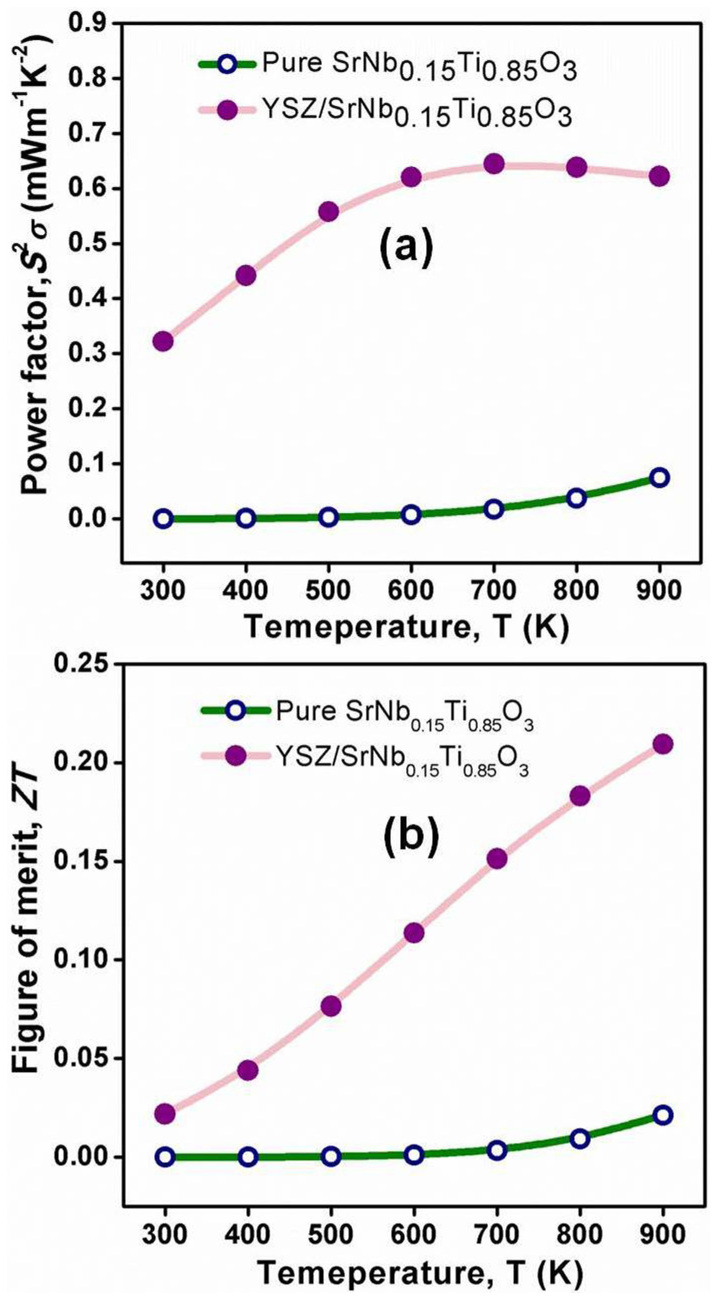
Temperature dependent thermoelectric power factor *S*^2^*σ* (a) and dimensionless figure of merit *ZT* (b) of YSZ/Nb-STO composite.

The thermoelectric power factor *S*^2^*σ* and dimensionless figure of merit *ZT* of the composite were shown in Fig. 3. The power factor was increased more than 7-fold as compared with the sample without YSZ inclusion (Fig. 3(a)), which is mainly beneficial from the increased electrical conductivity. Owing to the low thermal conductivity and high electrical conductivity, the *ZT* value is enhanced 9-fold, up to 0.21 at 900 K.

## Discussion

The carrier concentration (*n*), Hall mobility (*μ*), carrier effective mass (m*/*m_0_*) and m*/*n*^2/3^ at room temperature were summarized in the Table 1. With YSZ inclusion, the mobility was enhanced significantly, however, relatively, there was little change in the carrier concentration, which contributed to the enhancement of the electrical conductivity, according to the relation formula: 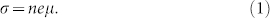
For degenerate semiconductors (parabolic band, energy-independent scattering approximation), the Seebeck coefficient (*S*) is given by^26^: 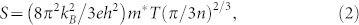
where *k*_B_ is Boltzmann constant and *h* is Planck constant. The equation indicates the Seebeck coefficient strongly depends on the value, m*/*n*^2/3^. Table 1 exhibited YSZ inclusion had very small impact on the value, m*/*n*^2/3^, which led to that Seebeck coefficient was almost independent of YSZ inclusion.

**Figure 4:**
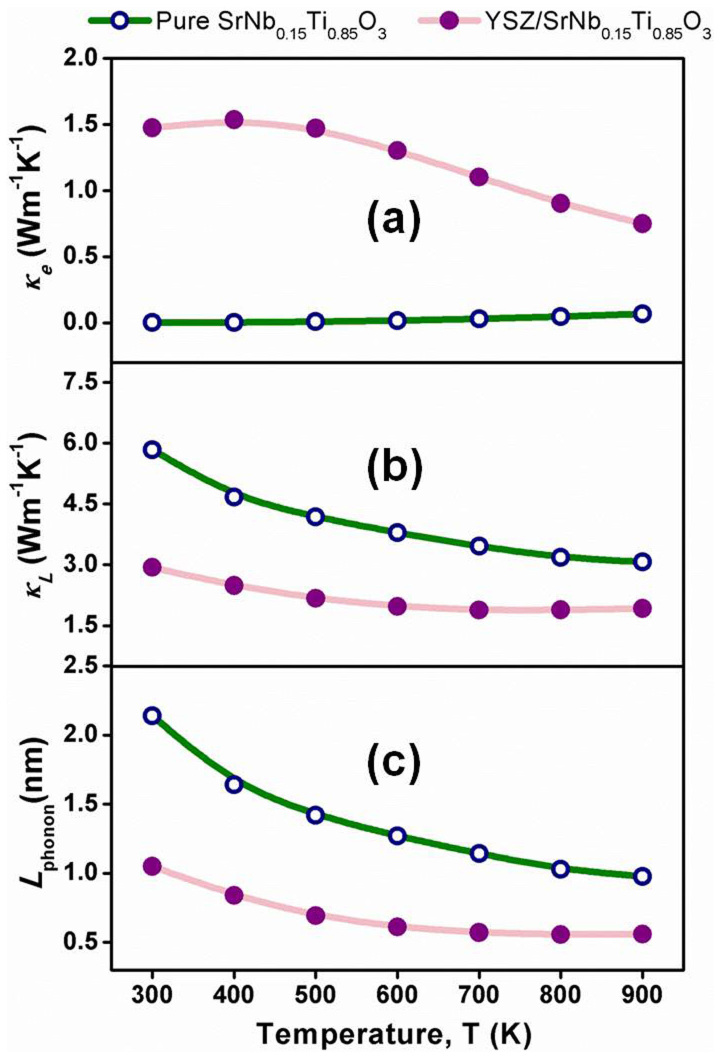
Temperature dependent electronic thermal conductivity (*κ*_e_) (a), lattice thermal conductivity (*κ*_L_) (b) and phonon mean free path (*L*_phonon_) (c) of YSZ/Nb-STO composite.

Temperature dependent electronic thermal conductivity (*κ*_e_), lattice thermal conductivity (*κ*_L_) and phonon mean free path (*L*_phonon_) of YSZ/Nb-STO composites were shown in Fig. 4. The *κ*_e_ was subtracted from the total thermal conductivity by using the Wiedemann-Franz law: 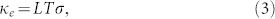
where *L* is the Lorenz factor, 2.45 × 10^−8^ V^2^/K^2^. The resulting *κ*_L_ was obtained by the relation: 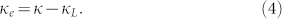
*L*_phonon_ was evaluated by the following equations^27^,^28^, 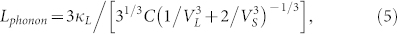
where *V*_L_, *V*_s_, *C*, were the longitudinal sound velocity, transverse sound velocity and specific heat capacity, respectively. Fig. 4(c) revealed that YSZ inclusion significantly decreased *L*_phonon_ and further induced the reduction in *κ*_L_ (Fig. 4(b)).

**Figure 5:**
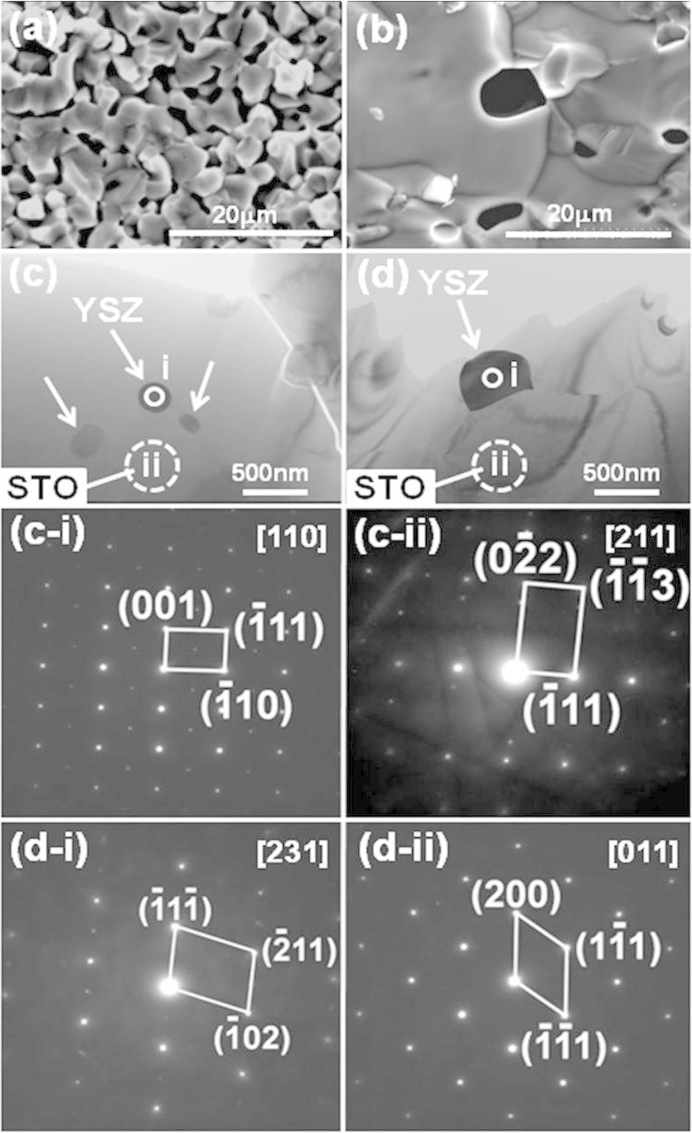
Microstructures and SAED patterns of YSZ/Nb-STO composite. (a) SEM of sample without YSZ inclusion; (b) SEM of sample with YSZ inclusion; (c) TEM of sample with YSZ inclusion inside grain; (d) TEM of sample with YSZ inclusion in triple junction; (c-i) SAED pattern taken from i-area in Fig. 5(c); (c-ii) SAED pattern taken from ii-area in Fig. 5(c); (d-i) SAED pattern taken from i-area in Fig. 5(d); (d-ii) SAED pattern taken from ii-area in Fig. 5(d).

**Figure 6:**
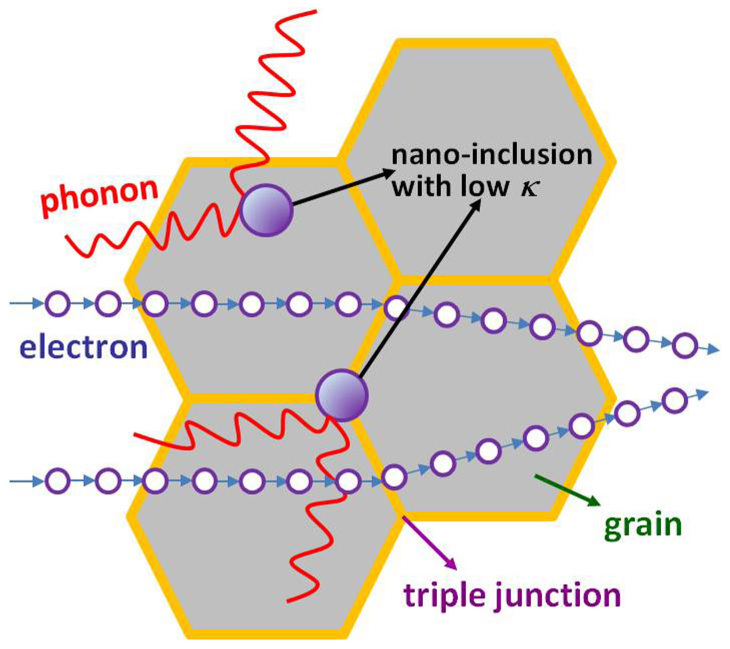
Schematic of phonon-nanoinclusion interaction and electron-nanoinclusion interaction.

Scanning electron micrographs (SEM) of YSZ/Nb-STO composite (Fig. 5(a) and (b)) showed the grain size was increased obviously by YSZ inclusion. To confirm where YSZ existed in bulk Nb-STO ceramic, transmission electron microscope (TEM) observation and selected area electron diffractions (SAED) were carried out. The SAED patterns in Fig. 5(c-ii) and Fig. 5(d-ii), taken from large crystalline area in Fig. 5(c) and Fig. 5(d), were only well-matched with a cubic strontium titanate crystal. However, the SAED patterns in Fig. 5(c-i) and Fig. 5(d-i), taken from black nano-sized particles in Fig. 5(c) and Fig. 5(d), revealed some diffraction spots except the diffraction spots of strontium titanate, which matched the diffraction spots of YSZ very well. Above-mentioned evidences fully proved that YSZ inclusions located inside the grain (Fig. 5 (c)) and in the triple junction (Fig. 5(d)), and these inclusions were nano-sized, from several tens nanometers to several hundreds nanometers. YSZ has much lower thermal conductivity, ~2.5 Wm^−1^K^−1^ below 1200 K^29^, compared with SrTiO_3_^30^.YSZ nano-inclusions inside the grain and in the triple junction could scatter the phonons effectively and thus brought about the decrease in the phonon mean free path, the lattice thermal conductivity and the total thermal conductivity, in sequence. As for SrTiO_3_ ceramic with oxide additives, such as TiO_2_, Al_2_O_3_ and SiO_2_, at the sintering temperature of over than 1400°C, the liquid phase formed at triple grain junctions and exhibited complete wetting of the grain boundaries^31^. YSZ/Nb-STO sample exhibited the abnormal grain growth in our experiment, which could be a similar liquid phase sintering behavior to SrTiO_3_ ceramic with added TiO_2_, Al_2_O_3_ and SiO_2_ in Ref. 31. According to the liquid-phase sintering theory proposed by Kingery^32^, the liquid phase formation could lead to the complete wetting of the grain boundaries, increase the grain boundary mobility and thus markedly accelerate the sintering rate and grain growth. The abnormal crystal growth of strontium titanate grain could obviously diminish the number density of grain boundaries and further reduce interface scattering of electrons remarkably, which partly contributed to the increased mobility and further to the enhanced electrical conductivity. On the other hand, Compared with pure Nb-STO, the relative density of YSZ/Nb-STO composite was increased from 63.1% to 79.4%, which was also partly contributed to the enhanced electrical conductivity. Fig. 6 showed the interaction schematic of phonon-nanoinclusion and electron-nanoinclusion. Oxide nano-inclusion with the low thermal conductivity, located inside the grain and in the triple junction, can reduce the thermal conductivity by effective interface phonon scattering, enhance the electrical conductivity by promoting the abnormal grain growth and increasing the relative density, and thus lead to the obvious enhancement of *ZT* value of Nb-STO.

In summary, YSZ nano-inclusion could effectively reduce the thermal conductivity and increase the electrical conductivity of Nb-STO, and thus obviously enhance the *ZT* value, which strongly suggested that oxide nano-inclusion with low thermal conductivity could be an effective strategy to enhance the thermoelectric properties of oxide thermoelectric materials with high thermal conductivity. Oxide nano-inclusion distributed inside the grain and in the triple junction can obviously reduce the phonon mean free path by the effective interface phonon scattering, and further apparently lead to the decrease in the thermal conductivity. Meanwhile, the oxide nano-inclusion with high surface activation can promote the grain growth, diminish the number density of grain boundaries, increase relative density and thus improve the carrier mobility and the electrical conductivity. Our research may give some helpful enlightenment to develop high-performance oxide thermoelectric materials.

## Methods

### Sample preparation

Commercial YSZ (8 mol% yttria-stabilized zirconia) particles with the size of 25 ~ 30 nm were used as an oxide inclusion. Nb-doped SrTiO_3_ ceramic sample with YSZ inclusion of 3 wt% were fabricated by the conventional normal pressure sintering method at 1500°C for 3 h in an Ar atmosphere. The relative densities of Nb-doped SrTiO_3_ ceramic without added YSZ and with YSZ inclusion of 3 wt% are 63.1% and 79.4%, respectively.

### Sample characterization

The thermoelectric performances, such as the Seebeck coefficient and electrical conductivity, were measured at 300–900 K in an Ar atmosphere by using an automatic thermoelectric measuring apparatus (Ozawa RZ2001K). The thermal diffusivity was measured by the usual laser flash method (ULVAC-RIKO TC-9000V). The carrier concentration and mobility was measured by the Van der Pauw's method (RESITEST 8300).The microstructures were observed on a scanning electron microscope (SEM) and a JEM-2010 transmission electron microscope (TEM). The specific heat capacity was measured by a differential scanning calorimeter system (TA Instrument DSC-2910). The mean phonon velocity was measured by an ultrasonic pulse-echo method (Panametrics-NDT 5800). The electrical properties (electrical conductivity and Seebeck coefficient) and thermal conductivity were measured in in-plane and cross-plane directions. YSZ/Nb-STO polycrystalline ceramic composite exhibited the isotropic electrical conductivity, Seebeck coefficient and thermal conductivity.

## Author Contributions

N.W. and K.K. designed the experiments. N.W. carried out the fabrication of materials and thermoelectric measurements. W.N. and M.K. contributed to microstructural characterizations. H.J.C. and H.C.H. provided helps in the experiments. N.W. and H.J.C. wrote the paper, and all authors reviewed the manuscript.

## Figures and Tables

**Table 1:** Carrier concentration (*n*), Hall mobility (*μ*), carrier effective mass (m*/*m*_0_, *m*_0_ = free electron mass) and m*/*n*^2/3^ at room temperature

	*n* (10^21^ cm^−3^)	*μ* (cm^2^V^−1^s^−1^)	m*/m_0_	m*/*n*^2/3^ (10^−14^ cm^2^)
Pure Nb-STO	1.57†)	8 × 10^−4^	1.75	1.29m_0_
YSZ/Nb-STO	2.06	6.11	2.16	1.27m_0_

^†)^estimated using the lattice constant and relative density (63.1%) of pure Nb-STO.
